# “Alert-Audit-Act”: assessment of surveillance and response strategy for malaria elimination in three low-endemic settings of Myanmar in 2016

**DOI:** 10.1186/s41182-018-0092-y

**Published:** 2018-04-13

**Authors:** Aye Mon Mon Kyaw, Soundappan Kathirvel, Mrinalini Das, Badri Thapa, Nay Yi Yi Linn, Thae Maung Maung, Zaw Lin, Aung Thi

**Affiliations:** 1 National Malaria Control Programme/Vector Borne Disease Control, Department of Public Health, Ministry of Health and Sports, Nay Pyi Taw, Myanmar; 2 International Union Against Tuberculosis and Lung Disease, Southeast Asia, New Delhi, India; 3 Department of Community Medicine, School of Public Health, Postgraduate Institute of Medical Education and Research, Chandigarh, India; 4 Médecins Sans Frontières (MSF) OCB, New Delhi, India; 5 World Health Organization Country Office for Myanmar, Yangon, Myanmar; 6 Department of Medical Research, Ministry of Health and Sports, Nay Pyi Taw, Myanmar

**Keywords:** Reactive case detection, Low endemicity, Indigenous malaria, Case notification, Case investigation, Operational research

## Abstract

**Background:**

Myanmar, a malaria endemic country of Southeast Asia, adopted surveillance and response strategy similar to “1-3-7” Chinese strategy to achieve sub-national elimination in six low-endemic region/states of the country. Among these, Yangon, Bago-East, and Mon region/states have implemented this malaria surveillance and response strategy with modification in 2016. The current study was conducted to assess the case notification, investigation, classification, and response strategy (NICR) in these three states.

**Methods:**

This was a retrospective cohort study using routine program data of all patients with malaria diagnosed and reported under the National Malaria Control Programme in 2016 from the above three states. As per the program, all malaria cases need to be notified within 1 day and investigated within 3 days of diagnosis and response to control (active case detection and control) should be taken for all indigenous malaria cases within 7 days of diagnosis.

**Results:**

A total of 959 malaria cases were diagnosed from the study area in 2016. Of these, the case NICR details were available only for 312 (32.5%) malaria cases. Of 312 cases, the case notification, investigation, and classification were carried out within 3 days of malaria diagnosis in 95.5% cases (298/312). Of 208 indigenous malaria cases (66.7%, 208/312), response to control was taken in 96.6% (201/208) within 7 days of diagnosis.

**Conclusion:**

The timeline at each stage of the strategy namely case notification, investigation, classification, and response to control was followed, and response action was taken in nearly all indigenous malaria cases for the available case information. Strengthening of health information and monitoring system is needed to avoid missing information. Future research on feasibility of mobile/tablet-based surveillance system and providing response to all cases including imported malaria can be further studied.

## Background

Malaria is still among the top 10 causes of mortality despite reduction by 48% of malaria incidence rate and 44% of mortality between 2010 and 2016 in the Southeast Asian region (SEAR) [1]. The Global Technical Strategy for Malaria 2016–2030 (GTS) calls for elimination of malaria in 10 countries by 2020 and 35 countries by 2030 [2].

Robust surveillance and response mechanism is necessary to accelerate and ensure malaria elimination which is identified as one of the three pillars under GTS framework [2, 3]. Surveillance assists the healthcare system to actively identify the unreported cases and also to identify the hidden infection source for further control of malaria [4]. Among the 14 countries of Asia Pacific Malaria Elimination Network (APMEN), 13 countries reported the availability of such surveillance system in place. Similarly, the member countries have reported about the malaria case investigations to all or part of cases notified depending on the burden and type of malaria cases (indigenous or imported malaria) [5].

China is one of the member countries of APMEN who successfully established a good surveillance and response system called as “1-3-7” strategy (case notification within 1 day, case investigation and classification within 3 days, and focus investigation and response within 7 days of malaria diagnosis) under its national program [6, 7]. The performance of 1-3-7 strategy in China was encouraging but suggested the need for proactive malaria hotspot mapping, assessment of genetic diversity, and population dynamics to eliminate malaria [6–9].

Myanmar, a malaria endemic SEAR country, has made significant progress in reducing malaria morbidity and mortality in 2016 compared to 2012 [4]. Despite these advancements, malaria remains to be the country’s major public health problem with annual parasite incidence of 3.5 per 1000 population and 21 deaths in 2016 [1]. The country has adopted similar malaria surveillance and response strategy like China with modifications to achieve sub-national elimination as per Myanmar National Plan for Malaria Elimination (NPME) 2016–2030 of National Malaria Control Programme (NMCP) [10]. It has been proposed to eliminate falciparum malaria from six low-endemic states and regions namely Yangon, Bago, Mon, Mandalay, Magway, and Nay Pyi Taw regions/states of Myanmar by 2020 and in the remaining nine regions/states by 2030. Among these, Yangon, Bago-East, and Mon regions/states have implemented this strategy in 2016, and the remaining states started implementing from 2017 [11].

Though the malaria surveillance and response strategy has been rolled out, assessment of this strategy has not yet been studied systematically. Assessment is needed to further review and strengthen the existing surveillance and response mechanism to accelerate the malaria elimination. Thus, this study was conducted to assess the malaria case notification, investigation, and response strategy in three low-endemic settings (Yangon, Bago-East, and Mon region/states) of Myanmar. The specific objectives were to assess the (a) number and proportion of patients diagnosed with malaria (stratified for socio-demographic and clinical characteristics), (b) number and proportion of patients with malaria notified within 1 day of diagnosis, (c) number and proportion of patients with malaria investigated and classified within 3 days of diagnosis, and (d) number and proportion of indigenous malaria cases for which the response to control action has been taken within 7 days of diagnosis.

## Methods

### Study design

This was a retrospective cohort study using electronic record review of routine program data collected under NMCP.

### General setting

The Republic of the Union of Myanmar, a WHO SEAR country, is administratively divided into 14 regions/states and Nay Pyi Taw Council Territory. Malaria is endemic in 291 out of 330 townships in Myanmar [12]. NMCP is carrying out malaria control and elimination activities, and the services are provided free of charge to the population. Basic health staff (BHS; health assistant, lady health visitor, midwives, and public health supervisors) of the formal public health system provides malaria diagnostic and treatment services at a facility level, and village health volunteers (VHV) provide the malaria control services at a community level with the special focus on rural, hard to reach areas.

### Specific setting

**Fig. 1:**
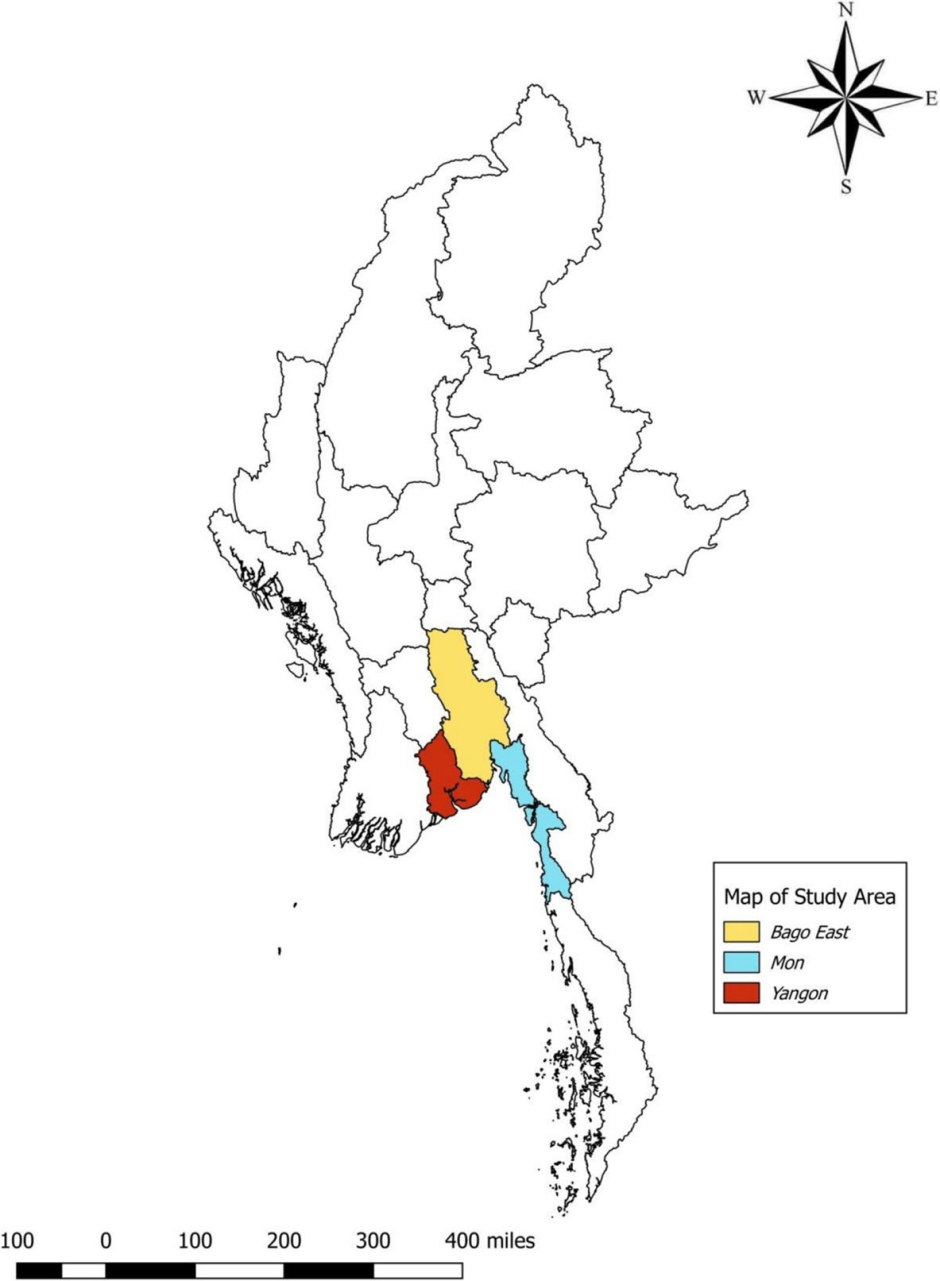
Map of Myanmar with study areas implemented the malaria surveillance and response strategy, 2016

Yangon (45 townships), Bago-East (14 townships), and Mon (10 townships) region/states (Fig. 1) of Myanmar have a population of 7.5, 2.95, and 2.1 million, respectively [10, 12]. The annual parasite index (API) of Yangon, Bago-East, and Mon region/states was 0.07, 0.86, and 2.25 in respectively in 2015 [13]. In total, all townships of Bago-East and Mon and 11/45 townships of Yangon are malaria endemic.

#### Case notification, investigation, classification (NIC), and response (NICR) strategy

Malaria NICR strategy in Yangon, Bago-East, and Mon region/states has been implemented since January 2016. As per the strategy, all malaria-positive cases must be notified within 24 h. Case investigation and classification of notified cases should be carried out using the standard investigation form within 3 days of diagnosis, and response to control should be taken within 7 days of malaria diagnosis. The foci investigation and response has not been started by the program which also needs to be carried out within 7 days.

**Fig. 2:**
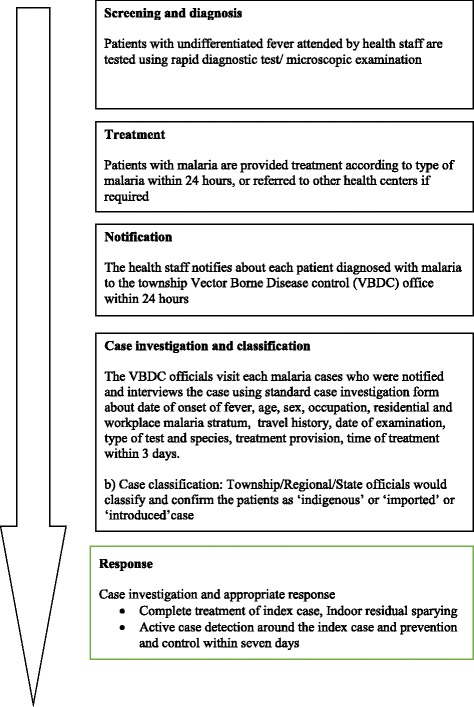
Case notification, investigation, classification, and response strategy in Yangon, Bago-East, and Mon region/states of Myanmar, 2016

Patients with undifferentiated fever are tested using a malaria dual antigen (both *Plasmodium vivax* and *Plasmodium falciparum*) rapid diagnostic test by VHV at the village level and BHS at facility/outreach. Once a malaria case is diagnosed by a VHV, it is informed to BHS by phone call. BHS further informs the township/regional vector borne disease control (VBDC) office again by phone call. Similarly, cases diagnosed by BHS are notified to VBDC office directly over phone. The township VBDC staff and health assistant at the township health department are informed further to carry out the case NIC. Once the case investigation and classification is done, the township VBDC team sends the filled form to regional/state VBDC officials. The regional/state VBDC team is asked to verify the case investigation and classification and suggests the appropriate response to be carried out in case of indigenous malaria. The case was classified as “indigenous” or “imported” or “introduced” based on the travel history and the link with imported malaria case. The process of case NICR is described in Fig. 2, and operational definitions NICR and malaria case classification are detailed in Table 1 [3, 11, 14].

**Table 1:** Operational definitions for malaria case notification, investigation, classification, and response strategy implemented in low-endemic settings of Myanmar, 2016 [3, 10, 11, 14]

Malaria stratum	The malaria stratum are classified as high (> 5/1000 population), moderate (1–5 case per 1000 population), low (< 1 case per 1000 population), and potential transmission and malaria free areas based on the annual parasite incidence, presence, or absence of indigenous cases, presence or absence of main vectors of malaria, and ecology. Each village is classified as malarious (stratum 3), potentially malarious (stratum 2), or malaria-free area (stratum 1). The malarious villages are further stratified into high risk (3a), moderate risk (3b), and low risk (3c).
Case notification	Compulsory reporting of detected cases of malaria by all medical units and medical practitioners, to either the health department or the malaria elimination service.
Case investigation	Collection of information to allow classification of a malaria case by origin of infection, i.e., whether it was imported, introduced, indigenous, or induced. Case investigation includes administration of a standardized questionnaire to a person in whom a malaria infection is diagnosed.
Response to control	Response to control refers to the response following the case investigation which consists of (a) active case detection through blood examination/symptom screening of the febrile cases in and around 10 households of the index cases and (b) vector control measures are IRS, distribution of LLINs (for positive cases), larval source management, and IEC/BCC activities.
Malaria case classification	
Indigenous case	A case contracted locally with no evidence of importation and no direct link to transmission from an imported case.
Imported case	Malaria case or infection in which the infection was acquired outside the area (outside region or state or country) in which it is diagnosed.
Introduced case	A case contracted locally, with strong epidemiological evidence linking it directly to a known imported case (first-generation local transmission).

#### Study population and period

All patients with malaria diagnosed and reported under NMCP between January and December 2016 in Yangon, Bago-East, and Mon region/states were included in the study.

#### Data variables and source of data

Details of all patients with undifferentiated fever screened for malaria by BHS and VHV are routinely collected in carbonless registers and sent to township VBDC office every month. The same is further sent to regional/state-level VBDC office. The data are entered in a national malaria compile database (Microsoft Office Excel) at the township/regional level by a data assistant. The socio-demographic (age, sex, region/state, type of healthcare provider) and clinical (type of test, malaria species, severity, and treatment status) characteristics of malaria cases diagnosed were obtained from the national malaria compile database.

There is no formal mechanism of maintaining the notified malaria cases, as all new cases are informed over phone with township VBDC staff. The filled case investigation forms submitted at the regional/state level are routinely entered into the case investigation database (Microsoft Office Excel) by the data assistant. There is no specific identifier (name or other unique identifier) available to match the data between the national malaria compile database and case investigation form. The day of case notification, case investigation, and classification from the day of diagnosis (categorized as first, third, or seventh day), and time of response to control (categorized as third or seventh day from diagnosis) and the type of response to control were collected.

#### Analysis and statistics

The electronic data entered in the national malaria compile database and case investigation database are separately exported to Statistical Package for Social Science (IBM Corp. Version 18) for analysis. Numbers and proportions of patients diagnosed with malaria, notified, and case-investigated within 1, 3, or 7 days of diagnosis, type of malaria classified (indigenous, imported, or introduced), and response to control carried out in indigenous malaria were calculated. Similarly, number and proportions are calculated for socio-demographic and clinical characteristics of patients diagnosed with malaria and case-investigated. Chi-square tests were performed to compare the proportions of the categorical variables. The *p* value < 0.05 was taken as statistically significant.

## Results

### Burden of malaria

A total of 959 (0.4%) of patients with undifferentiated fever were found malaria positive among 231,098 patients with fever, screened from Yangon, Bago-East, and Mon region/states. The annual parasite index (number of new parasitologically confirmed malaria cases per 1000 population at risk per year) of malaria cases detected under NMCP in 2016 for Yangon, Bago-East, and Mon was 0.01 (52/7,510,139), 0.14 (414/2,952,897), and 0.24 (493/2,096,099), respectively (data not tabulated).

### Characteristics of patients with malaria

Of 959 patients with malaria, 83.6% (802/959) were aged ≥ 15 years and 75.4% (723/959) were males. BHS diagnosed 77.7% (745/959) of malaria cases. *Plasmodium vivax* was the most common (55%, 436/793) species identified in these cases. The socio-demographic and clinical characteristics of patients diagnosed with malaria in these three areas are described in Table 2.

**Table 2:** Socio-demographic, clinical characteristics of patients with malaria in three low-endemic settings of Myanmar, 2016

Characteristics		All malaria		Malaria NIC information available		Malaria NIC information not available		*p* value
		*n*	(%)^a^	*n*	(%)^b^	*n*	(%)^b^	
Total		959		312		647		
State/region	Yangon	52	(5.4)	52	(100)	0	(0)	
	Bago-East	414	(43.2)	75	(18.1)	339	(81.9)	
	Mon	493	(51.4)	185	(37.5)	308	(62.5)	
Age (years)	0–14	157	(16.4)	59	(37.6)	98	(62.4)	0.17
	≥ 15	802	(83.6)	253	(31.5)	549	(68.5)	
Sex	Male	723	(75.4)	238	(32.9)	485	(67.1)	0.72
	Female	236	(24.6)	74	(31.4)	162	(68.6)	
	Pregnant^c^	5	(2.1)	na				
Type of healthcare provider	Basic health staff	745	(77.7)	271	(36.4)	474	(63.6)	< 0.001
	Village health volunteer	214	(22.3)	41	(19.2)	173	(80.8)	
Malaria species^d^	*Plasmodium vivax*	436	(55.0)	171	(39.2)	265	(60.8)	0.99
	*Plasmodium falciparum*	321	(40.5)	126	(39.3)	195	(60.7)	
	Mixed	35	(4.4)	15	(42.9)	20	(57.1)	
	*Plasmodium ovale*	1	(0.1)	–				
Severity	Uncomplicated	927	(96.7)	na		na		
	Complicated	32	(3.3)	na		na		

*NIC* case notification, investigation, and investigation; *Mixed* both *Plasmodium falciparum* and *Plasmodium vivax* positive; *na* information not available

^a^Column percentage

^b^Row percentage

^c^Percentage among total female

^d^Data for 166 patients among all malaria is not available

### NICR information availability

NICR details were available only for 312 (32.5%) malaria cases. For the remaining cases (67.5%, 647/959), neither electronic data from case investigation database nor hard copy of the case investigation form was available. The socio-demographic and clinical characteristics of the patients with malaria for whom NICR information is available are given in Table 2. Compared to the malaria cases for which the information is not available, the study did not miss cases systematically in any particular age group, sex, or malaria species (*p* > 0.05). However, the information of VHV-treated cases (80.8%, 173/2/214) were significantly missing (*p* < 0.001) compared to BHS-treated cases (63.6%, 474/745). The proportion of availability of NICR information in Yangon, Bago-East, and Mon region/states was 100% (52/52), 18.1% (75/414), and 37.5% (185/493), respectively. Among the NIC information available cases, 40.1% (125/312), 12.2% (38/312), 21.2% (66/312), and 13.1% (41/312) were forest goers, farmers, construction workers, and others (student/housewife/retired), respectively (data not tabulated).

### Case notification, investigation, classification (NIC), and response strategy (NICR)

**Fig. 3:**
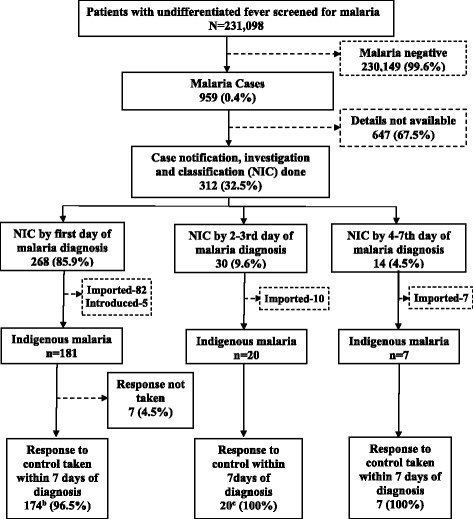
The surveillance and response strategy for malaria elimination in low-endemic settings of Myanmar, 2016

The flow of patients with malaria under NICR strategy in Yangon, Bago-East, and Mon state/regions is given in Fig. 3. The number of proportion of malaria cases notified with 24 h of diagnosis was not calculated since no such data on case notification was separately maintained. As the exact date/day of notification was not available, it was combined with case investigation and classification details and reported. For the available case information, the malaria case NIC were carried out within 3 days in 95.5% cases. For the remaining 4.5% of cases, NIC was carried out within 7 days (fourth to seventh day) of malaria diagnosis.

Among the total 312 cases, 66.7% (208/312), 31.7% (99/312), and 1.6% (5/312) cases were classified as indigenous, imported, and introduced malaria, respectively, as per operational definition. The response to control was taken in 96.6% (201/208) of cases within 7 days of malaria diagnosis of all indigenous malaria cases. In 34.8% (70/201) of cases, the response action was taken within 3 days of malaria diagnosis. During response to control, 1185 undifferentiated febrile patients were identified, and all were tested negative for malaria.

## Discussion

### Summary

The current study assessed the malaria NICR implemented for malaria elimination in Yangon, Bago-East, and Mon region/states of Myanmar in 2016. The NICR details were not available for nearly two thirds of cases. Among the malaria cases for which the information was available, case NIC were done within 3 days of malaria diagnosis in 95.5% of cases. Nearly two thirds of the investigated malaria cases were classified as indigenous and warranted response action. The response to control like mass symptom/blood screening, treatment of cases detected, and distribution of the insecticide impregnated bed nets was taken within 7 days in 96.6% of indigenous malaria cases. The results positively indicate that case NICR is being implemented as planned in Myanmar.

### Strengths

This is the first study conducted to assess the case NICR strategy under NMCP in Myanmar after the implementation. This could guide the program further to place standard operating procedures, build capacity of the health workers, improve the data quality, and strengthen the existing health system to prevent and control malaria. The study included all malaria cases diagnosed in Yangon, Bago-East, and Mon region/states from January 2016 to December 2016. The use of routinely collected programmatic data is one of the strengths of this study which reflects the field reality.

### Case notification within 1 day

The day/date of case notification is not maintained as it happens over phone. The date of diagnosis and notification should be collected to assess the timeliness of the surveillance system and to initiate the investigation of index malaria case for further response and control. Similarly, the BHS or VHV have to report the malaria cases twice first for the surveillance system over phone immediately after diagnosis of a malaria case and monthly by carbonless registers (paper based) to township VBDC office. It is to be noted that nearly two thirds of malaria cases are diagnosed at the community level in Myanmar which necessitates the strengthening surveillance at the community level using information and communication technology interventions [1, 7–9]. Establishment of real-time web-based health information system (HIS) in Myanmar will ensure the immediate availability of notification data and may reduce the workload and double reporting by BHS and VHV. NMCP has recently piloted mobile or tablet-based case NICR, and the results of the same are awaited.

### Case investigation and classification within 3 days

The proportion of malaria cases investigated and classified within in 3 days was 95.5% in Myanmar which aligns well with other countries in APMEN, such as China (85–100%), Indonesia (90.8%), and Thailand (86.7%) [7–9, 15]. This is a remarkable achievement for the Myanmar NMCP which has just started its sub-national elimination activities in 2016. Though case investigation and classification is possible within 1 day of diagnosis, the 86% achievement questions the adherence by VBDC staff to comprehensive steps involved in case investigation and classification as these are done by different teams. This can be due to case investigation of index case over telephone or at health facility immediately after the diagnosis.

As Myanmar is aiming for malaria (sub-national) elimination, classification of the cases needs careful attention. Misclassification of indigenous case as imported is possible as there is huge population mobility between borders of states/regions with heterogeneous malaria transmission in the country [9, 16, 17]. Similarly, case NICR for mobile, migrant population is one of the key challenges in effective implementation of surveillance strategy and in achieving elimination. This warrants mapping of the mobile and migrant clusters, target profiling, and strengthening the existing mobile, migrant follow-up units of the country to conduct active surveillance and response. It is also important to document previous episodes of malaria infection and necessary post-treatment follow-up to classify cases correctly for appropriate action [3].

### Response to control malaria by seventh day

The response to control like complete treatment of index case, indoor residual spray, reactive case detection (RACD) in family and neighborhood is well placed within the surveillance system. However, the foci investigation (identification, characterization, classification, and follow-up) and response are yet to be implemented in the study area which is one of the important components for sustained elimination. There is also a need to specify who should be tested during foci investigation in standard operating procedure (it is under development by the country), e.g., only symptomatic cases or all asymptomatic cases [3, 7, 9, 16].

High burden (67%) of indigenous malaria indicates the existing active transmission. As the elimination effort gains momentum, the regions/states tend to get more imported cases rather than indigenous cases and this is likely to flip in few years of time in these three regions/states. The imported malaria also warrants action in future following detailed investigation and classification if sustainable elimination is needed.

### Limitations

The findings of this study should be interpreted with caution in view of the following limitations. The case NIC details were not available for nearly two thirds of malaria cases diagnosed during the study period. The investigators made all their efforts to trace the forms (either in hard or soft copy) to extract the data but failed. However, except for cases diagnosed and treated by VHV, the study did not systematically miss cases with regard to age, sex, and malaria species compared to cases for which the information is missing. The high proportion of missing information of patients treated by VHV may be due to delayed involvement of VHV in surveillance, delayed reporting of cases, failure of reporting by VHV to BHS or BHS to township VBDC staff, or could be due to non-traceable cases especially in rural and remote areas.

The BHS and VHV primarily use rapid diagnostic test (RDT) kits rather than microscopic examination for diagnosis of cases in Myanmar. The primary and sole use of RDT for diagnosis may miss the low-density malaria infections and the elimination target [7, 9, 16]. Identification and notification of all malaria case is one of the rate limiting factors for malaria elimination; a dual diagnostic method, i.e., use of highly sensitive RDT and quality control mechanism, is needed. Simultaneous collection of blood smear in a sample/all patients tested with RDT and reconfirmation at township health facility could validate the findings. Similarly, review meetings at regular intervals to discuss sample of cases at the township or higher level could also validate the findings; in addition, it could improve the capacity of health workers.

Complete tracking of patients from fever to diagnosis, case notification, investigation, or response was not carried out as the national malaria compile database and case investigation databases could not be linked due to non-availability of a specific identifier. The RACD monitoring and evaluation tool is tested in China, Indonesia, and Thailand which can be adopted by Myanmar with modifications for the local needs.

#### Recommendations

Malaria case notification should be made mandatory at least in these three study regions/states by all healthcare providers namely private healthcare providers, informal health providers, military, and health departments other than public health departments. Uptake of RACD monitoring and evaluation tool and developing/adopting clear SOP specifying the responsibilities of health workforce at each level along with timeline is needed. Adequate capacity building of the staff is necessary based on the SOP. Development of web-based HIS linked with mobile phones may potentially improve the availability of information and coverage and speed up the response to control. At different levels, the reporting system should be linked by a unique identification number to get the complete details of a case and validation of the obtained data. Quality assurance strategies like reconfirmation of diagnosis using blood microscopy or other advanced diagnostic methods may be included in the SOP and implemented. Similarly, regular review meeting to discuss the cases at the township or higher level should be established. Gradually, the townships of neighbor regions/states should also be included for reporting to understand about the imported malaria. Myanmar should adopt best malaria elimination standard practices from other malaria-eliminated countries and neighboring countries which are in the verge of elimination and tailor to the existing program infrastructure and needs.

## Conclusion

The timeline at each stage of the strategy namely case notification, investigation, classification, and response to control was followed, and response action was taken in nearly all indigenous malaria cases for the available case information. Strengthening of health information and monitoring system is needed to avoid missing information. Future research on feasibility of mobile/tablet-based surveillance system and providing response to all cases including imported malaria can be further studied.

## Abbreviations

API: Annual parasite index
APMEN: Asia Pacific Malaria Elimination Network
BHS: Basic health staffs
CISDCP: China Information System for Disease Control and Prevention
GTS: Global Technical Strategy for Malaria 2016–2030
HIS: Health information system
NIC: Notification, investigation, and classification
NICR: Notification, investigation, classification and response strategy
NMCP: National Malaria Control Programme
NPME: Myanmar National Plan for Malaria Elimination 2016–2030
RACD: Reactive case detection
RDT: Rapid diagnostic test
SEAR: Southeast Asian region
SOP: Standard operating procedures
VBDC: Vector borne disease control
VHV: Village health volunteers
WHO: World Health Organization
